# Cluster Analysis of Motor Symptoms in Early-Diagnosed Parkinson’s Disease Patients

**DOI:** 10.3390/brainsci15050467

**Published:** 2025-04-28

**Authors:** Renee M. Hendricks, Shreyasi Biswas

**Affiliations:** School of Information Sciences, University of Illinois, Champaign, IL 61820, USA

**Keywords:** Parkinson’s disease, cluster analysis, patient clusters

## Abstract

Parkinson’s disease (PD) is a common movement disorder affecting adults. People diagnosed with PD can have a multitude of physical (motor) symptoms, including tremors, and rigidness, and psychological (non-motor) symptoms, including anxiety and depression. These symptoms dramatically affect daily living activities, including dressing oneself, preparing meals, and speaking and writing. Background/Objectives: To determine the symptom similarities and differences among PD patients, a method referred to as cluster analysis can be applied to patient data. This method can separate patients who differ by symptom presence while grouping patients with disease similarities. Previous PD cluster analysis studies provided patient groups that were defined by their age and disease duration—both numerical values—and excluded categorical values, such as patient gender, family history of the disease, and symptom presence. In addition, patient age and disease duration were limited in range in previous studies, providing a patient group that was too similar to divide into distinct clusters. Methods: This study utilized a decision tree cluster analysis method applied to categorical symptom data from PD patients. The applied cluster method automatically determines the number of clusters, reducing estimation errors, as many cluster analysis methods require the end user to estimate the number of clusters prior to applying cluster analysis. A post analysis of additional categorical and numerical variables was conducted, and this provided a means to describe the PD patient clusters in terms of gender, family history of PD, median age, disease duration, and symptom presence. The patient dataset utilized was accessed from the Parkinson’s Progression Markers Initiative (PPMI) website. Results and Conclusions: The cluster analysis results provided a means to describe seven PD patient subtypes based on motor symptom presence, with the largest PD patient cluster containing half of the patient sample, and these individuals had three of the motor symptoms present: bradykinesia, rigidity, and tremors.

## 1. Introduction

Parkinson’s disease (PD) is the most common movement disorder worldwide, affecting adults 60 years of age and older [1]. PD is a progressive disease attributed to the loss of neurons in the substantia nigra, with the nigral dopamine neurons greatly diminished and Lewy bodies present in the remaining neurons [1]. PD was first described by James Parkinson in 1817 as a disease consisting of resting tremors in An Essay on the Shaking Palsy [2]. In addition to resting tremors, other motor (physical) symptoms associated with PD are bradykinesia, rigidity, and postural instability. Bradykinesia is defined as slow movements and is considered the primary motor symptom of PD [3]. Rigidity is associated with a feeling of stiffness, and clinicians assess this by examining the resistance of a patient’s muscle against stretching [3]. Postural instability, a balance problem that usually appears in the later stages of the disease, can make PD patients unsteady when standing, and it increases their risk of falls [4]. In addition, PD is typically an asymmetrical disease, in which the motor symptoms usually affect one side of the body first [5].

In addition to motor symptoms, patients can experience unexplained non-motor symptoms for years prior to a diagnosis. These symptoms include an impaired sense of smell, constipation, rapid eye movement sleep disorder, fatigue, cognitive problems, anxiety, and depression [2].

The aim of this study was to apply a decision tree cluster analysis method to determine if PD patient subtypes could be identified and defined by categorical information, specifically motor symptom presence. This study included categorical values, including patient gender, family history of the disease, and symptom presence, whereas past studies excluded this information and focused on patient age and disease duration. It is important to apply and determine the features of importance to best describe and understand PD patients, their symptoms, and their disease progression, as age and disease duration may provide a limited understanding of the disease.

## 2. Materials and Methods

This section defines the cluster analysis method applied and the PD patient data utilized.

### 2.1. PD Cluster Analysis

The premise of cluster analysis is that given a set of individuals, each described by a set of measures, these individuals can be grouped into clusters such that the individuals within a cluster have similar measures and are unlike the individuals in other clusters [6]. In this study, patients were clustered via motor symptoms that were present or not present. Post analyses of the clusters provided additional descriptions of the clusters and further comparisons among clusters, including gender and symptom pairing.

The most commonly utilized clustering method is partitional cluster analysis. In partitional cluster analysis, data are divided into non-overlapping subsets, where each data instance is assigned to one subset [7]. In this study, each patient is assigned to one cluster. Partitioning methods are useful for bioinformatics applications where a fixed number of clusters is desired; however, a drawback of these methods is that a user specifies the number of clusters as an input parameter [8]. Estimating the number of clusters without expertise in the domain area of analysis can lead to inaccurate clusters that are not well separated.

A systematic review of PD patient cluster analysis research was conducted by the authors of [9]. The majority of studies included a variety of clinical scale scores for clustering. The clinical scores appear numerical, but they, in fact, are ordinal, categorical values. For example, a patient may be asked to rate the severity of their symptom(s) using a scale of 0 (none) to 4, with 4 representing the highest severity choice. The values are labels and are not measurable, numerical values, such as age.

Even though the scale scores are ordinal, these values were treated as numerical variables in previous cluster analysis studies, which is incorrect. In addition to clinical scale scores, patient age and disease duration were common variables included in previous cluster analysis studies, whereas categorical variables of gender, family history, symptom presence, and the side of the body affected by symptoms were excluded. The use of numerical values may lie in the partitioning clustering method applied, called K-means cluster analysis, which is applicable only to numerical values. In addition, a limitation of the K-means method is that the end user has to estimate the number of clusters prior to analysis. This estimation can be conducted in a multitude of ways, including using statistical analysis methods of calculating standard error values, plotting these error values against the number of clusters (K), and selecting the number of cluster(s) where the line starts to decrease in the plot. This creates a gap in determining the optimal number of clusters prior to analysis. Furthermore, past cluster results pointed to two to five PD patient clusters, but with age of onset and disease duration values overlapping, the clusters were not distinct [9].

In this study, a decision tree cluster analysis method was applied as defined by the authors of [10], where the clusters are automatically discovered; thus, we do not estimate nor input the number of clusters prior to applying cluster analysis. In this cluster approach, each variable is separated by its attributes, one at a time [10]. For example, a variable labeled gender may contain two attributes: male and female. The decision tree clustering method would divide the males into one subgroup and the females into a separate subgroup. The discovery tree method then continues until the last variable’s attributes are separated out [10]. Because the variable attributes are creating the splits, no attribute (characteristic or feature) will be placed in an incorrect cluster, and the objects in each cluster will have identical attributes. This results in the clusters containing a silhouette coefficient of 1.0 [10].

The silhouette coefficient provides an overall measure of the goodness of cluster results, with values ranging from −1 to +1. This value represents both the cohesion and separation of the resulting clusters [11]. A value close to −1 means the objects are poorly clustered with differing attributes in each cluster, and the clusters overlapping. A value close to +1 means the objects are tightly clustered with identical attributes and well separated from the objects in other clusters [12].

In this study, motor symptom variables, which are categorical variables, were utilized for clustering. Each motor symptom variable contained two attributes, 0 and 1. Zero referred to the motor symptom not present at diagnosis (absent). One referred to the motor symptom present at diagnosis.

Starting with the first motor symptom variable (tremor), there are two attributes that were separated (0 and 1). Next, the second motor symptom variable (rigidity) attributes were separated. Lastly, the third motor symptom variable (bradykinesia) attributes were separated. The clustering method was applied utilizing the Python 3.12 programming language, with the result visualizations created in Tableau. The dataset was explored next.

### 2.2. PD Patient Data

The Parkinson’s Progression Markers Initiative (PPMI) was launched in 2010 to create an open-access dataset to speed up scientific breakthroughs and new treatments [13]. PPMI baseline patient data were utilized for this study, which contained information on 1127 patients: 687 (61%) male patients and 440 (39%) female patients. The median disease duration among patients was 8 months, with a median patient age of 61 years. The patient age variable was the age of disease onset. For motor symptoms present at diagnosis, 856 patients had tremor symptoms, 811 patients had rigidity symptoms, 909 had bradykinesia symptoms, and 109 had postural instability symptoms. With the limited number of patients with postural instability symptoms, this symptom was excluded from the analysis.

In addition, 560 (53%) patients had motor symptoms that affected the right side of their body, 464 (44%) had motor symptoms that affected the left side of their body, and 32 (3%) patients had motor symptoms that affected their entire body. After the removal of patients with postural instability, patients affected on both sides of the body, and those with missing values, the number of patients was reduced to 1024.

The patient population for analysis now consisted of 623 (61%) male and 401 (39%) female patients, maintaining the same gender groups as the initial dataset. The median disease duration was the same as the initial dataset at 8 months, in addition to the same median patient age of 61 years. The age range of males was 19–84 years of age, with a similar age range for females at 19.6–83 years. The age range appears wide-ranging compared to previous studies, but only 5% of these patients were 40 years of age and younger.

In terms of a family history of PD, 65% of patients did not have a family history of the disease. For motor symptoms present at diagnosis, 793 patients had tremor symptoms, 774 patients had rigidity symptoms, and 848 patients had bradykinesia symptoms. This summary of patient data is displayed in Table 1 below.

## 3. Results and Discussion

This section explores and compares the discovered PD patient motor symptom clusters.

### 3.1. PD Patient Clusters

In this study, a decision tree cluster analysis method was applied, in which each variable is separated by its attributes as defined in [10]. In this study, the three motor symptom variables—tremor, rigidity, and bradykinesia—were utilized for clustering. Each motor symptom variable contained two attributes: 0 (not present at diagnosis) and 1 (present at diagnosis). Additional variables, including gender, a family history of PD, and the side of the body affected by symptoms, were utilized for the post analysis of the clusters.

For the 1024 patients, the clustering method created seven PD patient clusters, as displayed in Table 2 below. The patient clusters are listed per row. Reviewing the first row, this cluster (Cluster 1) contained 24 PD patients with one motor symptom present at diagnosis: bradykinesia. This was the second-smallest patient cluster. The smallest PD cluster (Cluster 2) contained seven patients. These patients had rigidity present at diagnosis. The largest cluster contained 521 PD patients, which consisted of over half (51%) of the total patient sample. The patients in this group had all three motor symptoms—tremor, rigidity, and bradykinesia—present at diagnosis, as noted by the digit one in each symptom column.

Each cluster will be explored next, starting with the first three clusters of PD patients who had one motor symptom present at diagnosis. Table 3 shows the results of these three patient clusters.

### 3.2. PD Patient Clusters with One Motor Symptom

Three of the clusters consisted of PD patients with one motor symptom present at diagnosis. These three clusters combined consisted of 138 (13.5%) of the study sample. As listed in Table 3 above, PD patients in Cluster 1 had bradykinesia symptoms present at diagnosis. This cluster consisted of 24 patients, the second largest one-symptom cluster. The median disease duration was 8.5 months, and the median patient age was 54 years. The male median age was 58 years, while the female median age was 52 years. Furthermore, 46% of patients did not have a family history of PD.

The PD patients in Cluster 2 had rigidity symptoms present at diagnosis. This smallest cluster consisted of seven patients, with a median disease duration of 28 months and a median age of 60 years. Among male patients, the median age was 63 years, while among female patients, it was 54.6 years. Furthermore, 57% of patients did not have a family history of PD.

PD patients in Cluster 3 had tremor symptoms present at diagnosis. This cluster was the largest one-symptom cluster, consisting of 107 patients, with 61 (57%) males and 46 (43%) females. The median disease duration in this cluster was 14 months, with a median age of onset of 63 years. Among male patients, the median age was 63.5 years, while among female patients, it was 61.5 years. Additionally, 60% of patients did not have a family history of PD.

Of these one-symptom clusters, Cluster 1 consisted of more females than males, albeit by two, whereas Clusters 2 and 3 consisted of more males than females. In addition, Cluster 1 had more patients with a family history of the disease, whereas the opposite was true for Clusters 2 and 3. In addition, Cluster 1 consisted of PD patients with the youngest median age of 54 years and shortest duration at 8.5 months, whereas Clusters 2 and 3 consisted of PD patients with similar median ages of 60 and 63.5 years and longer disease durations of 28 months (Cluster 2) and 14 months (Cluster 3). The side affected by PD motor symptoms was split across the right-hand and left-hand sides, with Cluster 3 consisting of more PD patients affected on the right-hand side than on the left-hand side of the body, contrary to the trend observed in Clusters 1 and 2.

Next, each of the three clusters in which PD patients had two motor symptoms present at diagnosis was explored. Table 4 below shows the results of these patient clusters.

### 3.3. PD Patient Clusters with Two Motor Symptoms

These three clusters combined consisted of 357 (35%) patients out of the study sample. Patients in Cluster 4 had rigidity and bradykinesia symptoms present at diagnosis. This cluster consisted of 192 patients, the largest cluster among the two-symptom clusters, with 112 (58%) males and 80 (42%) females. The median disease duration for this cluster was 6.5 months, and the median age of onset was 58 years. Furthermore, 63.5% of patients did not have a family history of PD.

Cluster 5 had tremor and bradykinesia symptoms present at diagnosis. This cluster consisted of 111 patients, with 59 (53%) males and 52 (47%) females. The median disease duration for this cluster was 7.9 months, and the median age of onset was 62 years. Furthermore, 75% of patients did not have a family history of PD.

Cluster 6 consisted of PD patients with rigidity and tremor symptoms present at diagnosis. This cluster consisted of 54 patients, with 40 (74%) males and 14 (26%) females. The median disease duration in this cluster was 6.6 months, with a median age of onset of 59 years. Additionally, 61% of patients did not have a family history of PD. The side affected by motor symptoms was reported as the right side in 36 patients (67%) and the left side in 18 patients (33%).

Of these two-symptom clusters, all clusters consisted of more male than female PD patients. In addition, all clusters consisted of more patients with no family history of PD than with a family history of the disease. Clusters 4 and 6 consisted of PD patients with similar median ages of 58 and 59 years, and similar median disease durations of 6.5 and 6.6 months, respectively, whereas Cluster 5 PD patients had a median age which was slightly higher at 62 years, with a median disease duration of 7.9 months. Overall, however, age and disease duration were similar across these three clusters.

The most common motor symptom pair present at diagnosis was rigidity and bradykinesia. This occurred in Cluster 4 with 192 PD patients, followed by motor symptom pairs comprising tremor and bradykinesia in 111 patients, with the least common motor symptom pair comprising rigidity and tremor in 54 patients. The side affected by PD motor symptoms was split across the right-hand and left-hand sides in Clusters 4 and 5, with Cluster 6 consisting of more PD patients affected on the right-hand side than on the left-hand side of the body.

Next, the one cluster of PD patients who had three motor symptoms present at diagnosis is explored. Table 5 below shows the results of this patient cluster.

### 3.4. PD Patient Cluster with Three Motor Symptoms

The PD patients in Cluster 7 had all three motor symptoms present at diagnosis: tremor, rigidity, and bradykinesia. This cluster consisted of 521 patients, with 334 (64%) males and 187 (36%) females. The median disease duration was 7 months, and the median age of onset was 61 years. Additionally, 67% of patients did not have a family history of PD, with 289 patients affected by motor symptoms on the right side (55.5%) and 232 (44.5%) patients affected by motor symptoms on the left side.

### 3.5. Summary of PD Patient Clusters

A summary of the seven PD patient cluster results can be viewed in Table 6 below. The largest one-motor symptom cluster was Cluster 3 with 107 patients, where tremors were present initially. The largest two-motor symptoms cluster was Cluster 4 with 192 PD patients, with rigidity and bradykinesia present initially. As noted earlier, the largest patient cluster was Cluster 7, with over half of the PD patient population sample in this cluster (521), and all three motor symptoms were present among these patients.

Six patient clusters contained more males than females, but Cluster 1 consisted of 2 more females (13) than males (11). In addition, this cluster had more patients with a family history of PD (by two patients), whereas the remaining clusters had more PD patients without a family history of the disease. One additional note about Cluster 1 is that, even though 13 patients had a family history of the disease, this was not based on the 13 female individuals that comprised the cluster; instead, the patients comprised of 6 females and 7 males.

The median ages of PD patients across clusters were within a limited range of 54 years to 63 years. In Cluster 2, PD patients had rigidity symptoms present at diagnosis and had the longest median disease duration at 28 months (over 2 years), whereas Cluster 4 patients had the shortest median duration of 6.5 months with two motor symptoms: rigidity and bradykinesia.

As noted previously, patients who were 40 years of age and younger comprised only 5% of the total patient population. Among these younger patients, 12 were female (25.5%), and 35 were male (74.5%), with a median age of 36 years. Of these younger patients, 58% had all three symptoms. The distribution of motor symptoms in this group showed that 13% experienced rigidity and bradykinesia, 4% experienced tremor and bradykinesia, and 58% experienced tremor and rigidity. Rigidity was the only motor symptom that did not occur alone in this younger population. In terms of a family history of PD, 42.5% of these younger patients had a family history of the disease.

Even though the analysis included information for over 1000 patients, a large sample for analysis, this dataset may not fully represent the PD population. In addition, the use of baseline, cross-sectional PD patient data, is a limitation, as this provides information for a single moment in time and does not contain any disease progression or symptom changes over time. Furthermore, the cluster analysis method was applied to categorical information, and including numerical and categorical information in the future may provide more detailed descriptions of patient subgroups. In addition, this study focused on motor symptom presence, as only motor symptom data were available, and including motor and non-motor symptom presence, in the future, may provide a better understanding of PD patient symptom clusters.

## 4. Conclusions

This study utilized a decision tree cluster analysis method applied to categorical motor symptom data from 1024 PD patients. The cluster analysis results provided a method for describing seven PD patient motor symptom subtypes. The largest PD patient cluster consisted of half of the patient sample, and these individuals had three motor symptoms present: bradykinesia, rigidity, and tremors.

Post analysis of the variables excluded from the clustering provided descriptive patient clusters, including gender-based, age-based, and family history-based clusters. The baseline patient data were accessed from the PPMI website. Future cluster analyses require non-motor and motor symptoms in order to provide a thorough understanding of all possible PD clusters based on symptoms.

Comparing the one-motor, two-motor, and three-motor symptom clusters of PD patients, excluding the motor symptoms present at diagnosis, many attributes were the same among all clusters, including median age, median disease duration, more males than females in the majority of clusters, and a limited number of PD patients with a family history of the disease. All these similarities may connect to the overall patient study population. This points to the need to utilize as much differentiated data as possible in order to understand how PD affects everyone.

A future approach might entail clustering familial Parkinson’s disease cases into subgroups based on specific genetic mutations and possibly gender, as both may be associated with distinct symptom patterns. These subgroups could then be compared to similarly clustered, sporadic PD patients to evaluate differences and similarities in gender, family history, gene mutations, and symptom patterns across all subgroups.

The manner in which PD clinical survey scores are utilized for quantifying progression is incorrect. There is an assumption that the survey scores are numerical, similar to patient age, but in fact, they are categorical and rank values. These values are summed to create a total survey score, which is also incorrect. Furthermore, published studies have calculated PD progression as the total survey score divided by disease duration; this is also incorrect [9].

A common question posted on PD patient forums is as follows: what do clinical survey scores mean? This points to the importance of providing interpretable and correct information to patients. A cluster description may assist with this in providing detailed yet interpretable information for each cluster subtype. For example, one cluster may be described as such, utilizing Cluster 7 results: PD patients in this cluster have three motor symptoms present at diagnosis (tremors, rigidity, and bradykinesia), with a twice-as-high percentage observed with respect to male patients and with the majority not having any family history of PD.

As previously noted, a limitation of this study is the use of baseline PD patient data, as this provides information for a single moment in time. There is an important need to understand and define PD progression accurately. Cluster analysis may be a method for accomplishing this, by clustering longitudinal PD patient data. The assumption is that, as patient symptoms and disease characteristics change, a patient may change clusters. The movement from one cluster to another cluster is a viable way to define and track PD progression. If PD patients can be defined by distinct, characteristic clusters, this may assist clinicians, researchers, patients, and their caregivers in accurately diagnosing the disease and understanding its pathways, as they would be intently looking for these symptoms and characteristics.

Lastly, the decision tree cluster analysis method was applied to a previous version of the PPMI dataset, utilizing categorical variables including symptom presence. Of the 412 PD patients in that study, the largest cluster consisted of patients with all three motor symptoms (tremors, rigidity, and bradykinesia), consisting of 50% (206 patients), with twice as many male patients compared to female patients [10]. The result in this study supports the previous study, as the largest cluster consisted of 521/1024 (51%) of the patient sample with the same three motor symptoms present, with twice as many male patients compared to female patients. In addition, the largest one-symptom cluster consisted of patients with tremors in the previous study, which aligns with this study. Furthermore, the largest two-symptom cluster consisted of patients with rigidity and bradykinesia in the previous study, which aligns with the results of this study [10]. These studies support a path forward for defining PD patient symptom clusters and trends.

Future research can include applying the decision tree cluster analysis method to categorical and numerical patient data, to evaluate these resulting patient clusters, and to compare and contrast with previous studies which included one type of data. There is also a need to include motor and non-motor symptoms in cluster analysis, to understand all disease symptoms. Furthermore, there is a need to validate the cluster method and cluster results with repeating applications on multiple datasets. In addition, future internal validation methods can include generating the corresponding silhouette coefficient plots for each cluster to further validate the result is +1. In addition, a longitudinal approach could provide a more comprehensive understanding of how patient subtypes evolve.

## Figures and Tables

**Table 1:** Parkinson’s disease (PD) patient information.

	Number of Patients
Total	1024
Rigidity	774
Tremor	793
Bradykinesia	848
Gender	
Female	401
Male	623
Family History	
Family History of PD	354
No Family History of PD	670
Side Affected by PD	
Left	464
Right	560

**Table 2:** PD patient motor symptom clusters.

Tremor	Rigidity	Bradykinesia	Number of Patients	Cluster
0	0	1	24	1
0	1	0	7	2
1	0	0	107	3
0	1	1	192	4
1	0	1	111	5
1	1	0	54	6
1	1	1	521	7

**Table 3:** PD patient clusters with one motor symptom present.

	Cluster 1	Cluster 2	Cluster 3
Number of Patients	24	7	107
Motor Symptoms	Bradykinesia	Rigidity	Tremor
Gender			
Female	13	3	46
Male	11	4	61
Family History			
Family with PD	13	3	43
No Family with PD	11	4	64
Side Affected by PD			
Right	10	3	60
Left	14	4	47

**Table 4:** PD patient clusters with two motor symptoms present.

	Cluster 4	Cluster 5	Cluster 6
Number of Patients	192	111	54
Motor Symptoms	Rigidity and Bradykinesia	Tremor and Bradykinesia	Rigidity and Tremor
Gender			
Female	80	52	14
Male	112	59	40
Family History			
Family with PD	70	28	21
No Family with PD	122	83	33
Side Affected by PD			
Left	91	54	18
Right	101	57	36

**Table 5:** PD patient cluster with three motor symptoms present.

	Cluster 7
Number of Patients	521
Motor Symptoms	Rigidity, Tremor, and Bradykinesia
Gender	
Female	187
Male	334
Family History	
Family with PD	173
No Family with PD	348
Side Affected by PD	
Left	232
Right	289

**Table 6:** PD patient clusters.

	Cluster 1	Cluster 2	Cluster 3	Cluster 4	Cluster 5	Cluster 6	Cluster 7
Number of Patients	24	7	107	192	111	54	521
Motor Symptoms	Bradykinesia	Rigidity	Tremor	Rigidity and Bradykinesia	Tremor and Bradykinesia	Rigidity and Tremor	Rigidity, Tremor, and Bradykinesia
Gender							
Male	11	4	61	112	59	40	334
Female	13	3	46	80	52	14	187
Family History							
Family with PD	13	3	43	70	28	21	173
No Family with PD	11	4	64	122	83	33	348
Side Affected by PD							
Right	10	3	60	101	57	36	289
Left	14	4	47	91	54	18	232
Median Disease Duration (Months)	8.5	28	14	6.5	7.9	6.6	7
Median Age of Onset (Years)	54	60	63	58	62	59	61

## Data Availability

Researchers can access data from the Parkinson’s Progression Markers Initiative (PPMI) by applying for access at Access Data Parkinson’s Progression Markers Initiative (ppmi-info.org).
